# Supplementary material for: Six-month mortality has decreased for patients with curative treatment intent for head and neck cancer in Sweden
Source: PLoS One. 2024 Apr 16;19(4):e0296534. doi: 10.1371/journal.pone.0296534 (PMC11020944; doi:10.1371/journal.pone.0296534)
Supplement: Attachment — Submitted filename: PONE-D-23-41780_reviewer_comments.pdf [file pone.0296534.s001.pdf]

PLOS ONE

Six-month mortality has decreased for patients with curative treatment for head and neck cancer in Sweden

--Manuscript Draft--

**Manuscript Number:**	PONE-D-23-41780
**Article Type:**	Research Article
**Full Title:**	Six-month mortality has decreased for patients with curative treatment for head and neck cancer in Sweden
**Short Title:**	Six-month mortality has decreased for patients with curative treatment for head and neck cancer in Sweden
**Corresponding Author:**	Charbél Talani<br>Linköpings universitet Hälsouniversitetet: Linkopings universitet Medicinska Fakulteten<br>Linköping, SWEDEN
**Keywords:**	Head and Neck cancer; Cancer epidemiology; Early Death; Early Mortality; oral squamous cell carcinoma; Oropharyngeal squamous cell carcinoma
**Abstract:**	Background: In general, survival outcome for head and neck cancer (HNC) patients has improved. However, mortality within six months after diagnosis with curative intent remains high. The aim of this study was to identify risk factors for early death among patients with curative treatment, and furthermore, to analyze whether the risk of early death changed over the recent years.<br>Material and Method: This real-world, population-based, nationwide study from the Swedish Head and Neck Cancer Register (SweHNCR) included 16,786 patients with curative treatment decision at the multidisciplinary tumor board from 2008 to 2020.<br>Results: During the study period a total of 618 (3.7%) patients with curative-intended treatment died within six months from diagnosis. Patients diagnosed between 2008–2012 had a six-month mortality rate of 4.7% compared to 2.5% for patients diagnosed 2017–2020, indicating a risk reduction of 53% ($p < 0.001$) for death within six months. The mean time to radiation therapy in the 2008–2012 cohort was 38 days, compared to 22 days for the 2017– 2020 cohort, ($p<0.001$). The mean time to surgery from diagnosis was 22 days in 2008–2012, compared to 15 days for the 2017–2020 cohort, ($p < 0.001$).<br>Females had a 20% lower risk of dying within six months compared to males ($p = 0.013$). For every year older the patient was at diagnosis, a 4.8% ($p < 0.001$) higher risk of dying within six months was observed. Patients with a WHO score of 1 had approximately 2.4-times greater risk of early death compared to WHO 0 patients ($p < 0.001$). The risk of early death among WHO 4 patients was almost 28 times higher than for WHO 0 patients ($p < 0.001$)<br>Conclusions: We found that the risk of early death decreased significantly from 2008 to 2020. During this period the mean time to the start of treatment, was significantly reduced both for surgery and oncological treatment regimes. Among patients with a curative treatment intention, increased risk of early death was associated with male sex, older age, advanced disease, increased WHO score, and a hypopharyngeal tumor site.
**Order of Authors:**	Charbél Talani
	Anders Högmo, MD, PhD
	Göran Laurell
	Antti Mäkitie
	Lovisa Farnebo, MD, PhD
**Additional Information:**	
**Question**	**Response**
**Financial Disclosure**	Yes

Powered by Editorial Manager® and ProduXion Manager® from Aries Systems Corporation

Enter a financial disclosure statement that describes the sources of funding for the work included in this submission. Review the submission guidelines for detailed requirements. View published research articles from *PLOS ONE* for specific examples.<br><br>This statement is required for submission and **will appear in the published article** if the submission is accepted. Please make sure it is accurate.<br><br>**Funded studies**<br>Enter a statement with the following details:<br>• Initials of the authors who received each award<br>• Grant numbers awarded to each author<br>• The full name of each funder<br>• URL of each funder website<br>• Did the sponsors or funders play any role in the study design, data collection and analysis, decision to publish, or preparation of the manuscript?<br><br>Did you receive funding for this work?	
Please add funding details.<br>as follow-up to "**Financial Disclosure**<br><br>Enter a financial disclosure statement that describes the sources of funding for the work included in this submission. Review the submission guidelines for detailed requirements. View published research articles from *PLOS ONE* for specific examples.<br><br>This statement is required for submission and **will appear in the published article** if the submission is accepted. Please make sure it is accurate.	The Östergötland Regional Research Council, SC-2018-00231-53, SC-2018-00231-46

Powered by Editorial Manager® and ProduXion Manager® from Aries Systems Corporation

**Funded studies**<br>Enter a statement with the following details:<br>• Initials of the authors who received each award<br>• Grant numbers awarded to each author<br>• The full name of each funder<br>• URL of each funder website<br>• Did the sponsors or funders play any role in the study design, data collection and analysis, decision to publish, or preparation of the manuscript?<br><br>Did you receive funding for this work?"	
Please select the country of your main research funder (please select carefully as in some cases this is used in fee calculation).<br>as follow-up to "**Financial Disclosure**<br><br>Enter a financial disclosure statement that describes the sources of funding for the work included in this submission. Review the submission guidelines for detailed requirements. View published research articles from *PLOS ONE* for specific examples.<br><br>This statement is required for submission and **will appear in the published article** if the submission is accepted. Please make sure it is accurate.<br><br>**Funded studies**<br>Enter a statement with the following details:<br>• Initials of the authors who received each award<br>• Grant numbers awarded to each author<br>• The full name of each funder<br>• URL of each funder website<br>• Did the sponsors or funders play any role in the study design, data collection and analysis, decision to publish, or preparation of the manuscript?<br><br>Did you receive funding for this work?"	SWEDEN - SE
**Competing Interests**	The authors have declared that no competing interests exist.

Use the instructions below to enter a competing interest statement for this submission. On behalf of all authors, disclose any competing interests that could be perceived to bias this work—acknowledging all financial support and any other relevant financial or non-financial competing interests.<br><br>This statement is required for submission and **will appear in the published article** if the submission is accepted. Please make sure it is accurate and that any funding sources listed in your Funding Information later in the submission form are also declared in your Financial Disclosure statement.<br><br>View published research articles from *PLOS ONE* for specific examples.<br><br>**NO authors have competing interests**<br><br>Enter: *The authors have declared that no competing interests exist.*<br><br>**Authors with competing interests**<br><br>Enter competing interest details beginning with this statement:<br><br>*I have read the journal's policy and the authors of this manuscript have the following competing interests: [insert competing interests here]*<br><br>* typeset	
**Ethics Statement**<br><br>Enter an ethics statement for this submission. This statement is required if the study involved:<br><br>• Human participants<br>• Human specimens or tissue<br>• Vertebrate animals or cephalopods<br>• Vertebrate embryos or tissues	Ethics Committee approval; Gothenburg, number 299–14, T230-17 and Lund 2020-01972. Data was used from the Swedish Head and Neck cancer registry which is completely anonymous. The authors did not have access to information that could identify individual participants during or after data collection.

Powered by Editorial Manager® and ProduXion Manager® from Aries Systems Corporation

• Field research<br><br>Write "N/A" if the submission does not require an ethics statement.<br><br>General guidance is provided below. Consult the submission guidelines for detailed instructions. **Make sure that all information entered here is included in the Methods section of the manuscript.**	

Powered by Editorial Manager® and ProduXion Manager® from Aries Systems Corporation

**Format for specific study types**<br><br>**Human Subject Research (involving human participants and/or tissue)**<br>• Give the name of the institutional review board or ethics committee that approved the study<br>• Include the approval number and/or a statement indicating approval of this research<br>• Indicate the form of consent obtained (written/oral) or the reason that consent was not obtained (e.g. the data were analyzed anonymously)<br><br>**Animal Research (involving vertebrate animals, embryos or tissues)**<br>• Provide the name of the Institutional Animal Care and Use Committee (IACUC) or other relevant ethics board that reviewed the study protocol, and indicate whether they approved this research or granted a formal waiver of ethical approval<br>• Include an approval number if one was obtained<br>• If the study involved *non-human primates*, add *additional details* about animal welfare and steps taken to ameliorate suffering<br>• If anesthesia, euthanasia, or any kind of animal sacrifice is part of the study, include briefly which substances and/or methods were applied<br><br>**Field Research**<br><br>Include the following details if this study involves the collection of plant, animal, or other materials from a natural setting:<br>• Field permit number<br>• Name of the institution or relevant body that granted permission	
**Data Availability**<br><br>Authors are required to make all data underlying the findings described fully available, without restriction, and from the time of publication. PLOS allows rare exceptions to address legal and ethical concerns. See the PLOS Data Policy and FAQ for detailed information.	No - some restrictions will apply

A Data Availability Statement describing where the data can be found is required at submission. Your answers to this question constitute the Data Availability Statement and **will be published in the article**, if accepted.<br><br>**Important:** Stating 'data available on request from the author' is not sufficient. If your data are only available upon request, select 'No' for the first question and explain your exceptional situation in the text box.<br><br>Do the authors confirm that all data underlying the findings described in their manuscript are fully available without restriction?	
**Describe where the data may be found in full sentences. If you are copying our sample text, replace any instances of XXX with the appropriate details.**<br><br>• If the data are **held or will be held in a public repository**, include URLs, accession numbers or DOIs. If this information will only be available after acceptance, indicate this by ticking the box below. For example: *All XXX files are available from the XXX database (accession number(s) XXX, XXX.).*<br>• If the data are all contained **within the manuscript and/or Supporting Information files**, enter the following: *All relevant data are within the manuscript and its Supporting Information files.*<br>• If neither of these applies but you are able to provide **details of access elsewhere**, with or without limitations, please do so. For example:<br><br>*Data cannot be shared publicly because of [XXX]. Data are available from the XXX Institutional Data Access / Ethics Committee (contact via XXX) for researchers who meet the criteria for access to confidential data.*<br><br>*The data underlying the results presented in the study are available from (include the name of the third party*	All relevant data are within the manuscript and its Supporting Information files.

and contact information or URL).<br>• This text is appropriate if the data are owned by a third party and authors do not have permission to share the data.<br><br>* typeset	
Additional data availability information:	

Powered by Editorial Manager® and ProduXion Manager® from Aries Systems Corporation

Manuscript
Click here to access/download;Manuscript;Talani et al Final.docx

Six-month mortality has decreased for patients with curative treatment for head and neck cancer in Sweden

Charbél Talani[1,2], Anders Högmo[3], Göran Laurell[4], Antti Mäkitie[5,6,7], Lovisa Farnebo[1,2]

1 Faculty of Medicine and Health Sciences, Department of Biomedical and Clinical Sciences, Division of Sensory Organs and Communication, Linköping University
2 Region Östergötland Anaesthetics, Operations and Specialty Surgery Center, Department of Otorhinolaryngology
3 Regional Cancer Center Western Sweden, Sahlgrenska University Hospital, Gothenburg, Sweden
4 Department of Surgical Sciences, Uppsala University, Uppsala, Sweden
5 Department of Otorhinolaryngology – Head and Neck Surgery, University of Helsinki and Helsinki University Hospital, Helsinki, Finland
6 Division of Ear, Nose and Throat Diseases, Department of Clinical Sciences, Intervention and Technology, Karolinska Institutet and Karolinska Hospital, Stockholm, Sweden
7 Research Program in Systems Oncology, Faculty of Medicine, University of Helsinki, Helsinki, Finland

Corresponding author

Charbél Talani
Universitetssjukhuset I Linköping, 58185 Linköping, Sweden
+46101032522
Charbel.talani@regionostergotland.se

Keywords: Head and neck cancer, Early mortality, Oral squamous cell carcinoma, Cancer epidemiology, Early Death

Brief description:
*The risk of early death decreased significantly from 2008 to 2020.
*Time from diagnosis to treatment decreased between 2008 to 2020.
*Among patients with a curative treatment intention, increased risk of early death was associated with male sex, older age, advanced disease, increased WHO score, and a hypopharyngeal tumor site.

Ethical Considerations: The Regional Ethical Review Board in Gothenburg and Lund reviewed and approved the study, (Gothenburg, number 299–14, T230-17 and Lund 2020-01972). This study was carried out in accordance with the current Helsinki Declaration of the World Medical Association from 2013. Data are available upon request.

Abstract

Background: In general, survival outcome for head and neck cancer (HNC) patients has improved. However, mortality within six months after diagnosis with curative intent remains high. The aim of this study was to identify risk factors for early death among patients with

curative treatment, and furthermore, to analyze whether the risk of early death changed over the recent years.

Material and Method: This real-world, population-based, nationwide study from the Swedish Head and Neck Cancer Register (SweHNCR) included 16,786 patients with curative treatment decision at the multidisciplinary tumor board from 2008 to 2020.

Results: During the study period a total of 618 (3.7%) patients with curative-intended treatment died within six months from diagnosis. Patients diagnosed between 2008–2012 had a six-month mortality rate of 4.7% compared to 2.5% for patients diagnosed 2017–2020, indicating a risk reduction of 53% (p <0.001) for death within six months. The mean time to radiation therapy in the 2008–2012 cohort was 38 days, compared to 22 days for the 2017–2020 cohort, (p<0.001). The mean time to surgery from diagnosis was 22 days in 2008–2012, compared to 15 days for the 2017–2020 cohort, (p <0.001).
Females had a 20% lower risk of dying within six months compared to males (p =0.013). For every year older the patient was at diagnosis, a 4.8% (p <0.001) higher risk of dying within six months was observed. Patients with a WHO score of 1 had approximately 2.4-times greater risk of early death compared to WHO 0 patients (p <0.001). The risk of early death among WHO 4 patients was almost 28 times higher than for WHO 0 patients (p <0.001)

Conclusions: We found that the risk of early death decreased significantly from 2008 to 2020. During this period the mean time to the start of treatment, was significantly reduced both for surgery and oncological treatment regimes. Among patients with a curative treatment intention, increased risk of early death was associated with male sex, older age, advanced disease, increased WHO score, and a hypopharyngeal tumor site.

Background

Head and neck cancer (HNC) is one of the most common cancers worldwide, and epidemiological assessments have reported a recent global increasing incidence[1-5], in Sweden as well as elsewhere over the recent years[6, 7]. Simultaneously, a reduced mortality has been noted in HNC in recent decades, due to better available treatments, less smoking, a multidisciplinary approach to treatment, and earlier discovery of tumors[8-11].

Numerous studies have dealt with survival in HNC patients, but population-based studies focusing on early death in HNC patients are scarce. Early death from HNC was previously defined as patients dying within six months of diagnosis in a study from the Swedish head and neck cancer register (SweHNCR)[12]. It reported that 4.5% of patients died within six months of diagnosis between 2008–2015, even though they received curative-intended treatment[12]. Curative-intended treatment is often aggressive, costly, and comes with considerable side effects. If patients receiving aggressive treatment die within six months, the physicians involved need to address whether the indications and decision-making involved in treatment management should be re-evaluated. Both surgery and radiotherapy, the backbones of head and neck cancer treatment, warrant repeated visits to the hospital and frequent hospitalization. Knowing the factors identifying patients at risk of early death could help clinicians better select suitable treatments, and thus avoid mutilating surgery and aggressive chemoradiotherapy in cases with a considerable risk of early death.

Since 2008, major progress in clinical management of patients with HNC has been made in Sweden. The care has, in general, been influenced by international initiatives aiming to improve quality of care of patients with HNC[5, 7]. Therefore, to study the efficacy of the new guidelines in an era of Human Papilloma Virus (HPV) and demographic changes, a further analysis of patients dying within six months of treatment is warranted. In this large,

real-world, nationwide, population-based study containing 18,739 patients (16786 curative and 1769 palliative) with HNC, the primary aim was to identify patients at risk of early death after treatment with curative intent, and to analyze factors that contribute to this increased risk. Furthermore, we wanted to test the hypothesis as to whether six-month mortality has decreased for HNC patients over the past few years.

Material and Methods

Ethics statement

Data were obtained from the SweHNCR (Ethics Committee approval; number 2020-01972). The SweHNCR is funded by the Swedish government and includes 98.5% of all HNC patients since 2008 when cross referenced with the register of the Department of Health and Welfare in Sweden. The authors did not have access to information that could identify individual participants during or after data collection. Application for data extraction was sent to Regional Cancer Centre Väst 15 October 2021. Decision of disclosure was made 12 November 2021, and the data file was delivered on 26 November 2021 (Number: SV2803, Diary number: HS2021-01178).

The total number of consecutively-affected Swedish patients during the period January 1, 2008–December 31, 2020 in the SweHNCR with at least six months of follow-up was 18,739[5].

Patient population

Patients with a palliative treatment decision at multidisciplinary tumor board (MDT) (n =1953, 10.4%) were not included in the study, leaving 16,786 patients with curative treatment intent for further analysis. Patients with curatively intended treatment were divided into three groups based on year of diagnosis; 2008-2012, 2013-2016, and 2017-2020 (Figure 1).

Ten sites of tumors were included: lip (C00.0–2, C00.6, C00.8, C00.9); oral cavity (C00.3, C00.4, C02, C03, C04, C05, C06); oropharynx (C01.9, C05.1, C05.2, C05.8, C05.9, C09, C10); nasopharynx (C11); hypopharynx (C12, C13); larynx (C10.1, C32); nose (C30.0) and nasal sinuses (C31); salivary glands (C07, C08); head and neck cancer of unknown primary (C77.0); and middle or inner ear (C 30.1). Malignant tumors located in the esophagus, thyroid, or parathyroid glands were not included. Data reported to the SweHNCR included: diagnosis, TNM classification (according TNM 7[13]), stage, sex, age, WHO score at diagnosis, time to treatment, treatment, follow-up, recurrence and survival. The Eastern Cooperative Oncology Group score, the WHO score, runs from 0 to 5, with 0 denoting perfect health and 5 death[14]. The WHO score was rated 0–4, indicating the physical performance status of the patient. A higher score indicates worse physical performance.

Figure 1. Flow chart describing treatment intent, year of diagnosis and early death for included patients.

HNC treatment in Sweden is centralized to seven university hospitals, although a few regional hospitals provide non-surgical oncological treatment. National Healthcare and Social Security systems are offered equally to all Swedish inhabitants. All patients are examined by either an oncologist or a head and neck surgeon after termination of treatment to evaluate treatment outcome, every three months for the first two years post treatment and every sixth months for the following three years.

Statistics

Results are presented as the mean, standard deviation, and range for continuous variables, and as numbers and percentages for categorical variables. Incidence rates for 2008-2020 and

incidence rate ratio was calculated comparing 2020 to 2008 with 95% confidence intervals according to a method described by Martin and Austin[6, 15-17]. Data from Statistics Sweden were used for information regarding yearly population count. SweHNCR was used to identify the number of new HNC cases annually. For comparisons between two groups (divided by; age, days to treatment, and days from diagnosis to death), the independent Student's t-test was used. ANOVA test (with Bonferroni method as a post hoc test), the chi-square test for categorical variables (stage, site, age groups, time periods, and WHO function class) and Fisher's exact test was used for tables with dichotomous variables (Sex, TNM score, and death). Exact binomial confidence intervals were estimated forproportions. Logistic regression analysis was used to control for confounding factors. To describe overall crude survival and early death for subgroups, a Kaplan-Meier plot was used, and the difference between subgroups (sex, time periods, age groups, and WHO class) was analyzed with a log-rank test. Cox regression was used for multivariable analyses with death within six months from diagnosis as failure. All significance tests were two-tailed and conducted at a 5% significance level. For all statistical analyses, we used IBM SPSS Statistics for Macintosh, Version 28.0. Armonk, NY: IBM Corp Released 2021.

Results

The annual number of new patients with HNC steadily increased from 2008 (n=1211 cases) to 2020 (n=1673 cases). During the same period, the population increased by 12%, from 9.2M to 10.4M, indicating that factors other than a growing population were involved in the increase of new HNC cases. We noted an incidence rate ratio of 23.8 % (CI 95% 23.5-24.2) between 2008 (13.0 diagnosed with HNC per 100000) and 2020 (16.1 diagnosed with HNC per 100000), corresponding to an annual incidence increase of 0.25 per 100000 ($p<0.001$) ~~(calculated as described in statistics section above)~~ (Figure 2). Both oropharyngeal and oral

cavity cancer displayed increased occurrence: the number of patients with oropharyngeal cancer rose with 86% from 2008 (n =270) to 2020 (n =503); oral cancer 48% from 2008 (n =343) to 2020 (n =509). The incidence rate ratio for oropharyngeal cancer was 66.1% (CI 95% 64.3-68.0) between 2008 (2.92 per 100000) and 2020 (4.85 per 100000), and for oral cavity cancer with 32.3% (CI 95% 32.0-33.6) from 2008 (3.71 per 100000) to 2020 (4.90 per 100000). All other sites had fairly similar occurrence over the years 2008-2020 (Figure 2). The number of patients discussed at multidisciplinary tumor boards (MDT) increased from 2008 to 2020 (95% to 99.5%, mean for the total study period 97%).

During the years 2008 to 2020, 618 (3.7%) of 16,786 patients died within six months, despite having a recommendation for curatively intended treatment at MDT (Figure 1).

Figure 2a. Annual number of new cases of head and neck cancer in Sweden per year 2008-2020
Figure 2b. Incidence of head and neck cancer in Sweden 2008-2020
Figure 2c. Distribution of tumor sites of head and neck cancer in Sweden 2008-2020

Predictors for six-month mortality

Year of diagnosis: The total cohort was divided into three groups based on the year of diagnosis. The three groups contained a comparable number of patients: 2008–2012 (n=5778), 2013–2016 (n=5307), and 2017–2020 (n=5701) (Table 1, and 2). Between 2008–2012, six-month mortality was 4.7% compared to 3.9% and 2.5% for the years 2013–2016 and 2017–2020, respectively ($p < 0.001$) (Table 1, Figure 3). Sex, age, and stage were evenly distributed throughout the three groups. The 2008–2012 group had a slightly higher distribution of unknown WHO scores compared to the two latter groups, $p < 0.001$ (Table 2). The composition of oropharyngeal and oral cavity cancer differed between the 2017–2020 cohort (30.5% and 28.3% of all HNC cancer respectively) compared to the 2008–2012 cohort (25.3% and 26.3% respectively), $p < 0.001$.

Figure 3. Kaplan-Meier curve of death within six months from diagnosis based on year of diagnosis

Table 1. Descriptive data for patients with curative treatment intent, and univariate analysis of death within 6 months.

N =16786	Prevalence n (%)	Dead within 6 months/N (%)	HR (95% CI)	P-values
**Sex**				
Male	10,712 (63.8)	424/10,712 (4.0)	1.00	-
Female	6074 (36.2)	194/6074 (3.2)	0.80 (0.68–0.95)	0.012
**Total**	16,786(100)	618/16,786(3.7)	-	-
**Age** continuous (mean (sd))	66.1 (12.6)			
**Age groups**				
18–39	463 (2.8)	0/463 (0)	-	-
40–49	1139 (6.8)	14/1139 (1.2)	1.00	-
50–59	3043 (18.1)	52/3043 (1.7)	1.40 (0.77-2.52)	0.268
60–69	5378 (32.0)	155/5378 (2.9)	2.37 (1.37-4.10)	0.002
70–79	4368 (26.0)	203/4368 (4.6)	3.86 (2.24-6.62)	<0.001
80+	2395 (14.3)	194/2395 (8.1)	6.88 (4.00-11.8)	<0.001
**WHO Score**				
0	12,078 (72.0)	206/12,078 (1.7)	1.00	-
1	2244 (13.4)	145/2244 (6.5)	3.88 (3.14–4.80)	<0.001
2	890 (5.3)	108/890 (12.1)	7.53 (5.97–9.51)	<0.001
3	321 (1.9)	68/321 (21.2)	14.4 (11.0–19.0)	<0.001
4	36 (0.2)	16/36 (44.4)	37.2 (22.4–62.0)	<0.001

Missing	1217 (7.3)	75/1217 (12.1)	3.72 (2.85–4.84)	<0.001
**Year of diagnosis**	16,786 (100)	618/16,786 (3.7)	-	-
2008–2012	5778 (34.4)	273/5778 (4.7)	1.00	
2013–2016	5307 (31.6)	205/5307 (3.9)	0.82 (0.68–0.98)	0.026
2017-–2020	5701 (34.0)	140/5561 (2.5)	0.51 (0.42-0.63)	<0.001

Table 2. Descriptive data for patients with curative treatment intent grouped by time of diagnosis and univariate analyses

Variable	2008–2012	2013–2016	2017–2020	Total N (%)	Sig. p-value
Oropharynx N (%)	1461 (25.3)	1499 (28.2)	1738 (30.5)	4698 (100)	<0.001
Oral Cavity N (%)	1531 (26.5)	1514 (28.5)	1615 (28.3)	4660 (100)	<0.001
Other sites N (%)	2786 (48.2)	2294 (43.2)	2348 (41.2)	7428 (100)	<0.001
Male N (%)	3713 (64.3)	3416 (64.4)	3583 (62.8)	10,712 (100)	0.174
Female N (%)	2065 (35.7)	1891 (35.6)	2118 (37.2)	6074 (100)	0.173
WHO 0 N (%)	4005 (69.3)	3780 (71.2)	4293 (75.3)	12078 (72.0)	0.013
WHO 1 N (%)	720 (12.5)	763 (14.4)	761 (13.3)	224 (13.4)	1.00*
WHO 2 N (%)	289 (5.0)	287 (5.4)	314 (5.5)	890 (5.3)	0.544*
WHO 3 N (%)	108 (1.9)	95 (1.8)	118 (2.1)	321 (1.9)	0.246*
WHO 4 N (%)	14 (0.2)	12 (0.2)	10 (0.2)	36 (0.2)	0.604*
WHO missing N (%)	642 (11.1)	370 (7.0)	205 (3.6)	1217 (7.3)	<0.001*
Total patients N (%)	5778 (100)	5397 (100)	5791 (100)	16,786 (100)	-

Time to Surgery, mean days***	22	19	14	19**	<0.001
Time to Radiation therapy, mean days***	38	30	22	29**	<0.001

*Chi-square between WHO1 and other WHO scores

**Mean duration in days between 2008–2020

***Days from diagnosis to start of treatment

Time to treatment: Time to the start of treatment from diagnosis was shorter in the 2017–2020 group compared to the group 2008–2012. The mean time to radiotherapy/chemoradiation therapy in the 2008–2012 cohort was 38 days, compared to 22 days for the 2017– 2020 cohort, p<0.001. The mean time to surgery from diagnosis was 22 days in 2008–2012, compared to 15 days for the 2017–2020 cohort, p <0.001 (Table 2).

Sex: Altogether, 10,712 (63.8%) of patients were male, and 6074 (36.2%) were female (Table 1). A significant difference in mortality between the sexes was found. The six-month mortality rate among male patients was 4.0% compared to 3.2% for females (p =0.012) (Table 1) (Figure 4). Thus female patients had a 20% lower risk of death within six months compared to male patients.

Figure 4a. Kaplan-Meier curve of death within six months from diagnosis based on sex
Figure 4b. Kaplan-Meier curve of death within six months from diagnosis based on age at diagnosis
Figure 4c. Kaplan-Meier curve of death within six months from diagnosis based on WHO function class at diagnosis

Age: The mean age at diagnosis was 66 years (range: 18–102). Males were on average one year younger than the females (65.7 versus 66.7) at diagnosis. The risk of dying within six months increased with age. For every year older the patient was at diagnosis, six-month mortality increased by 4.8% (p <0.001). No patient below 40 years of age died within six months, compared to 8.1% of all patients older than 80 years (p <0.001). There was a 6.88-fold increased risk of dying within six months for patients over 80 years of age compared to patients below 50 years of age (Table 1) (Figure 4).

WHO score: 12,078 (72%) of all patients had a WHO score of 0. Only 1.7% of all patients with WHO 0 died within six months, compared to 44.4% of those with WHO 4 (p <0.001). In univariate analysis, the risk of death within six months was 37.2 times higher for patients with WHO 4 compared to those with WHO 0 (Table 1). Mean survival time among patients dying within six months was 119 days for WHO 0 patients compared to 76 days for WHO 4 patients (p =0.004) (Figure 4).

Site: The most common tumor site was the oropharynx (28%) followed by the oral cavity (27.8%) and larynx (12.7%) (Table 3). The six-month mortality rate varied between tumor sites. Worst outcomes were seen in patients with hypopharyngeal cancer, with 10.3% dying within six months despite curative treatment intent. A univariable analysis showed that patients with a hypopharyngeal tumor had an almost 2.5-fold higher relative risk of dying within six months compared to oropharyngeal patients (p <0.001) (Table 3).

Table 3. Descriptive data for patients with curative treatment intent, and univariate analysis of death within 6 months.

N =16786	Prevalence n (%)	Dead within 6 months/N (%)	HR (95% CI)	P-value
**Tumor site**				
Oropharynx	4698 (28.0)	146/4698 (3.1)	1.00	-
Oral Cavity	4660 (27.8)	201/4660 (4.3)	1.40 (1.12–1.73)	0.002
Larynx	2134 (12.7)	89/2134 (4.2)	1.35 (1.04–1.76)	0.026
Hypopharynx	591 (3.5)	61/591 (10.3)	3.45 (2.56–4.65)	<0.001
Nasopharynx	381 (2.3)	5/381 (1.3)	0.42 (0.17–1.02)	0.056
Sino Nasal	690 (4.1)	26/690 (3.8)	1.22 (0.80–1.85)	0.355
Salivary Gland	1212 (7.2)	35/1212 (2.9)	0.93 (0.64–1.34)	0.683
Lip	1850 (11.0)	35/1850 (1.9)	0.61 (0.42–0.88)	0.008
CUP	559 (3.3)	19/559 (3.4)	1.10 (0.68–1.77)	0.705
Ear canal	11 (0.1)	1/10 (10)	3.46 (0.48–24.7)	0.216
**Stage**				
I	4948 (29.5)	73/4948 (1.5)	1.00	-
II	2847 (17.0)	66/2847 (2.3)	1.58 (1.13–2.20)	0.007
III	2077 (12.4)	87/2077 (4.2)	2.88 (2.11–3.93)	<0.001
IV A	5507 (32.8)	293/5507 (5.3)	3.68 (2.85–4.75)	<0.001
IV B	553 (3.3)	56/553 (10.1)	7.12 (5.03–10.1)	<0.001
IV C	93 (0.6)	14/93 (15.1)	11.0 (6.23–19.6)	<0.001
Missing	761 (4.5)	-	-	-
**T**				

T1-2	11,649 (69.4)	229/11,649 (2.0)	1.00	-
T3-4	4968 (29.6)	383/4968 (7.7)	4.04 (3.43–4.76)	<0.001
Missing	169 (1.0)	-	-	-
**N**				
N-	10,263 (61.2)	300/10,263 (2.9)	1.00	-
N+	6520 (38.8)	318/6520 (4.9)	1.68 (1.44–1.97)	<0.001
**M**				
M0	16,511(98.4)	596/16,511 (3.6)	1.00	
M1	102 (0.6)	15/102 (14.7)	4.36 (2.61–7.28)	<0.001
Missing	173 (1.0)	-	-	-

Stage: An association between tumor stage and six-month mortality was found. Among all patients in the total cohort, 54% were diagnosed with stage III or stage IV cancer (Table 3), although 55% of all women were diagnosed in stage I-II compared to 45% of all males (p<0.001). Of the 618 patients who died within 6 months, 76% had stage III or IV disease compared to 12% of patients with stage I. Significant differences in six-month mortality were also noted in stage IV. For stage I patients, six-month mortality was 1.5%, compared to 5.3%, 10.1%, and 15.1% for stages IV A, IV B, and IV C, respectively (p <0.001). In total, a patient with stage IV C disease had a 10-times higher risk of dying within six months compared to stage I patients (Table 3).

TNM class: Higher T class correlated with worse prognosis. Seventy percent of all patients had a T1-2 tumor at diagnosis (Table 3). Only 2% of the patients with a T1-2 tumor died within six months compared to 7.7% of T3-4 patients. We found that a patient with T3-4 tumor had a 3 times higher risk of death within six months compared to T1-2 patients (p <0.001) (Table 3).

A total of 11,649 (61%) of all patients had no neck metastasis (N-) at diagnosis, whereas 6520 (39%) had one or more metastases in the neck at diagnosis (N+). The six-month mortality for

N– patients was 2.9%, compared to 4.9% for N+ patients (p <0.001). In total, an N+ patient had a 1.68-fold higher risk of dying within six months compared to a N– patient (Table 3). One-hundred and two (0.6%) of all included patients had distant metastasis (M1) and were still considered at the MDT to benefit from curative treatment. However, 3.6% of patients with M0 disease died within six months, compared to 14.7% of patients with a M1 disease (p <0.001). We found that M1 patients had a 4.36-times higher risk of dying within six months compared to M0 patients (Table 3).

Independent factors for death within six months after diagnosis

A multivariable Cox regression was carried out with death within six months as the dependent variable (Table 4). Patients diagnosed between the years 2008–2012 had a six-month mortality rate of 4.7% compared to 2.5% for patients diagnosed between 2017–2020, indicating a risk reduction of 53% (p <0.001) for death within six months (Table 4). Females had a 20% lower risk of dying within six months compared to males (p =0.013). For every year older the patient was at diagnosis, a 4.8% (p <0.001) higher risk of dying within six months was observed. A 67-year-old patient had a 4.8% higher risk of dying within six months than a patient who was 66 years old.

Patients with a WHO score of 1 had approximately 2.4-times greater risk of early death compared to WHO 0 patients (p <0.001). The risk of early death among WHO 4 patients was almost 28 times higher than for WHO 0 patients (p <0.001).

A patient with a tumor localized in the hypopharynx had a 1.58-times higher risk of dying within six months compared to a patient with a tumor in the oropharynx (p =0.003). Patients with a T3/4 tumor had a 2.9-times higher risk of dying within six months compared to T1/2 patients (p <0.001). It was found that patients with neck metastasis at diagnosis had a 1.6-times higher risk of dying within six months compared to patients without nodal involvement

(p <0.001). Distant metastasis at diagnosis gave a 2.3-times higher risk of dying within six months (p =0.002) (Table 4).

These results form a risk profile where year of diagnosis, higher age, male sex, hypopharyngeal tumor, advanced TNM class, and higher WHO score constitute risk factors for early death.

Table 4. Multivariable Cox regression with death within six months as target variable for HNC patients undergoing curative-intended treatment.

Factor	HR (95% CI)	Significance
**Year of diagnosis**		
2008–2012	-	-
2013–2016	0.83 (0.69–1.00)	0.052
2017–2020	0.47 (0.40–0.61)	<0.001*
**TNM Score**		
T1-2 vs 3-4	2.90 (2.43–3.51)	<0.001*
N+/0	1.60 (1.36–1.99)	<0.001*
M+/0	2.30 (1.36–3.85)	0.002*
**Sex**	.800 (0.67–0.95)	0.013*
**Age Continuously**	1.05 (1.04–1.06)	<.001*
**WHO Function**		
WHO 0	-	-
WHO 1	2.38 (1.90–2.97)	<0.001*
WHO 2	3.84 (2.99–4.91)	<0.001*
WHO 3	7.87 (5.90–10.5)	<0.001*
WHO 4	27.6 (16.5–46.2)	<0.001*
WHO Missing	2.90 (2.21–3.81)	<0.001*
**Tumor Site**		
Oropharynx	-	-
Oral cavity	1.21 (0.95–1.53)	0.121
Larynx	1.13 (0.85–1.51)	0.397
Hypopharynx	1.58 (1.16–2.14)	0.003*
Nasopharynx	0.510 (0.21–1.24)	0.137
Sino nasal	0.984 (0.63–1.53)	0.943
Salivary Gland	0.890 (0.61–1.31)	0.555

Lip	0.629 (0.41–0.95)	0.029*
CUP	1.58 (0.95–2.64)	0.077
Ear	4.84 (0.67–34.9)	0.118

Discussion

This real-world, population-based, nationwide study, including 16,786 patients with HNC and curative treatment intent displayed that year of diagnosis, higher age, higher TNM class, male sex, higher WHO class, and a tumor in the hypopharynx were independent risk factors for death within six months of diagnosis. The effects of standardized protocols to ensure that all patients receive adequate treatment without unnecessary delays might have contributed to a significantly reduced risk of early death over recent years.

Throughout the study period, patients with curative treatment intent (n =16,786) had a 3.7% six-month mortality overall. Jensen et al. reported a 7.1% six-month mortality rate in a Danish nationwide study including 11,419 HNC patients between 2000–2017 treated with curatively intended radio- or chemoradiotherapy. The same author studied a series of 2209 HNC patients between 2010–2017 from multiple centers and found a non-cancer specific six-month mortality of 4.4% following treatment with radiotherapy[18, 19]. Nieminen et al. reported a six-month mortality of 11.4% in a subgroup of 317 HNC patients undergoing microvascular reconstruction[20]. As patients with HNC comprise a heterogenous population, the early mortality rate differs if subpopulations of patients are analyzed. Studies looking merely at treatment with radiotherapy, or at microvascular reconstruction contain selected patient cohorts likely affecting the rate of early death. Our study includes all surgical and oncological treatment options for patients with curative treatment intent and all T classes. Patients undergoing treatment with a free flap and microvascular reconstruction typically have at least a T2 tumor, indicating a disease with a higher risk of early death.

In the present study, men had a higher risk of early death, in accordance with the findings by Kouka et al. in their series of 8288 German HNC patients[21]. Other studies have not shown a sex-dependent rate of early death in HNC patients[12, 18]. In this study there is an uneven distribution between sexes regarding tumor stage and site; for example only 23.5% of hypopharyngeal cancer patients were female, which can partly explain the better prognostic outcome for females.

Other studies have suggested that estrogen could have a beneficial impact on survival after HNC [22-25], however that correlation was not analyzed here. In consistency with other studies [8, 12, 18, 20, 21, 26, 27], we saw that higher age and performance status correlated with an increased rate of early death. Furthermore, a hypopharyngeal tumor location, higher stage, and advanced T class were confirmed as significant independent risk factors for six-month mortality, in accordance with previous findings[12, 26, 28, 29].

Our study has revealed that the six-month mortality decreased throughout the period of 2008–2020. The reasons are most likely multifactorial. Sweden implemented standardized diagnostic work ups in 2015, which led to faster diagnoses and shortened waiting times until treatment for HNC patients. The same year, national guidelines for HNC treatment were published, leading to a national standardized consensus regarding diagnostic work up and treatment[30]. Furthermore, oropharyngeal cancer derived from HPV is increasing [2, 7, 31-33], and has a better prognosis compared to HPV-negative tumors[34, 35]. The substantial increase in HPV-positive cancers and the decreased prevalence of daily smokers in Sweden (12% in 2008 to 8% in 2020), contributing to less HPV-negative HNC cases can be part of the explanation as to why six-month mortality is decreasing for HNC patients [36-38].

It is reasonable to speculate that a number of variables not registered in the SweHNCR have improved the diagnostic work-up, treatment, and care of patients with HNC. Neck dissection was implemented 2015 in Sweden as the standard treatment for oral cavity cancer patients

with a depth of invasion of the primary tumor exceeding 3 millimeters and N0 status. This change is likely contributing to more active treatment of occult metastasis, and therefore to improved survival rates. It is also likely that new innovative therapy has had an impact on early mortality. Since 2008, there have been several improvements in oncological treatments such as the change of external radiotherapy from using 3D conformal radiotherapy to intensity-modulated radiotherapy and volumetric modulated arc radiotherapy, shifts from neoadjuvant high-dose cisplatin regimens to concomitant cisplatin. Concomitant cetuximab treatment was proven to be less effective, and was excluded from routine treatment[39]. New diagnostic tools have been increasingly available for accurate HNC diagnosis. The implementation of sentinel node biopsies in 2018 could also have contributed to identifying more contra- or bilateral metastases. Moreover, the use of FDG-PET/CT has improved the detection of regional and distant metastases. Altogether, this study suggests that standardized and rapid diagnostic work up and treatment, together with other factors, contributes to fewer HNC patients dying within six months of diagnosis.

Weaknesses of the study includes the fact that important parameters influencing overall survival, such as comorbidities, alcohol consumption, HPV status, and smoking habits were not recorded in the early years of the SweHNCR. Actual cause of death was not reported in the register, along with data on occupational exposures, socioeconomic factors, oral hygiene, complications to treatments, and gene mutations; all factors which could influence early mortality[1, 27, 40, 41].

Compensating for several of the study's limitations is the large population of 16,786 consecutive HNC patients with curative treatment intent, and the homogeneity of healthcare provided to all Swedes. The results in this study can be used to identify HNC patients at high risk of early death and thus open up for discussions about de-escalation of treatment for

selected cases to provide the best possible remaining time for patients at high risk of early death.

Conclusion

This first European population-based analysis of trends in six-month mortality among HNC patients with curative treatment intent is based on registry data comprising 16,786 patients. The study provides generalizable epidemiological data on early mortality and survival of curative HNC patients. The mean time to treatment was significantly decreased between the years 2008-2012 compared to 2017-2020. Encouragingly, the present results show that early mortality has decreased from 4.7% (2008–2012) to 2.5% (2017–2020) in Sweden. Female patients had a 20% lower risk of death within six months compared to male patients. Increased age at diagnosis, male sex, higher WHO function class, advanced TNM class, and tumor in the hypopharynx were independent risk factors for death within six months.

Acknowledgements: We would like to thank Mats Fredriksson, statistician at Linköping University, for excellent statistical guidance.

Conflict of interest: The authors declare no conflicts of interest.

References

1. Cohen N, Fedewa S, Chen AY. Epidemiology and Demographics of the Head and Neck Cancer Population. Oral Maxillofac Surg Clin North Am. 2018;30(4):381-95. Epub 20180803. doi: 10.1016/j.coms.2018.06.001. PubMed PMID: 30078696.

2. Roman BR, Aragones A. Epidemiology and incidence of HPV-related cancers of the head and neck. J Surg Oncol. 2021;124(6):920-2. Epub 20210923. doi: 10.1002/jso.26687. PubMed PMID: 34558067; PubMed Central PMCID: PMCPMC8552291.

3. Rettig EM, D'Souza G. Epidemiology of head and neck cancer. Surg Oncol Clin N Am. 2015;24(3):379-96. Epub 2015/05/17. doi: 10.1016/j.soc.2015.03.001. PubMed PMID: 25979389.
4. Gormley M, Creaney G, Schache A, Ingarfield K, Conway DI. Reviewing the epidemiology of head and neck cancer: definitions, trends and risk factors. Br Dent J. 2022;233(9):780-6. Epub 20221111. doi: 10.1038/s41415-022-5166-x. PubMed PMID: 36369568; PubMed Central PMCID: PMCPMC9652141.
5. Cancercentrum R. Swedish Head and Neck Cancer Register (SweHNCR) http://www.Cancercentrum.se: RCC, 2018.
6. Cancercentrum R. Årsrapport Nationellt Kvalitetsregister 2020. Cancercentrum.se: 2020.
7. samverkan RCi. Årsrapport nationellt kvalitetsregister, 2022. Cancercentrum: RCC; 2021.
8. Guo K, Xiao W, Chen X, Zhao Z, Lin Y, Chen G. Epidemiological Trends of Head and Neck Cancer: A Population-Based Study. Biomed Res Int. 2021;2021:1738932. Epub 20210714. doi: 10.1155/2021/1738932. PubMed PMID: 34337000; PubMed Central PMCID: PMCPMC8294963.
9. Gronhoj C, Hjalgrim L, Jakobsen KK, Charabi B, Mirian C, Laier GH, et al. Incidence of head and neck cancer in children: A Danish nationwide study from 1978 to 2014. Pediatr Blood Cancer. 2018;65(7):e27037. Epub 2018/04/01. doi: 10.1002/pbc.27037. PubMed PMID: 29603576.
10. Gatta G, Botta L, Sanchez MJ, Anderson LA, Pierannunzio D, Licitra L, et al. Prognoses and improvement for head and neck cancers diagnosed in Europe in early 2000s: The EUROCARE-5 population-based study. Eur J Cancer. 2015;51(15):2130-43. Epub 20150926. doi: 10.1016/j.ejca.2015.07.043. PubMed PMID: 26421817.
11. Mroueh R, Haapaniemi A, Grenman R, Laranne J, Pukkila M, Almangush A, et al. Improved outcomes with oral tongue squamous cell carcinoma in Finland. Head Neck. 2017;39(7):1306-12. Epub 20170508. doi: 10.1002/hed.24744. PubMed PMID: 28481417.
12. Talani C, Makitie A, Beran M, Holmberg E, Laurell G, Farnebo L. Early mortality after diagnosis of cancer of the head and neck - A population-based

nationwide study. PLoS One. 2019;14(10):e0223154. Epub 2019/10/03. doi: 10.1371/journal.pone.0223154. PubMed PMID: 31577831.
13. Sobin LH, Gospodarowicz MK, Wittekind C. TNM classification of malignant tumours. 7th 2009. ed. Chichester, West Sussex, UK ;: Wiley-Blackwell; 2010.
14. Oken MM, Creech RH, Tormey DC, Horton J, Davis TE, McFadden ET, et al. Toxicity and response criteria of the Eastern Cooperative Oncology Group. Am J Clin Oncol. 1982;5(6):649-55. Epub 1982/12/01. PubMed PMID: 7165009.
15. Befolkningsdatabas [Internet]. Statistics Sweden. 2022. Available from: https://www.statistikdatabasen.scb.se/pxweb/sv/ssd/START__BE__BE0101__BE0101A/BefolkningR1860N/table/tableViewLayout1/.
16. Statistical Database [Internet]. 2022.
17. Martin DO, Austin H. Exact estimates for a rate ratio. Epidemiology. 1996;7(1):29-33. doi: 10.1097/00001648-199601000-00006. PubMed PMID: 8664397.
18. Jensen KH, Vogelius I, Kristensen CA, Andersen E, Overgaard J, Eriksen JG, et al. Early Mortality after Radical Radiotherapy in Head and Neck Cancer - A Nationwide Analysis from the Danish Head and Neck Cancer Group (DAHANCA) Database. Clin Oncol (R Coll Radiol). 2021;33(1):57-63. Epub 20200719. doi: 10.1016/j.clon.2020.07.004. PubMed PMID: 32698963.
19. Jensen KH, Vogelius IR, Bernsdorf M, Gothelf A, Kristensen CA, Maare C, et al. Early non-cancer mortality risk prediction after curative-intent radiotherapy or chemoradiotherapy for head and neck squamous cell carcinoma. Radiother Oncol. 2022;171:1-6. Epub 20220405. doi: 10.1016/j.radonc.2022.03.020. PubMed PMID: 35395275.
20. Nieminen T, Tolvi M, Lassus P, Wilkman T, Lehtonen L, Makitie A. Risk factors for evaluating early mortality after microvascular reconstruction of head and neck cancers. Scand J Surg. 2022:14574969221117010. Epub 20220824. doi: 10.1177/14574969221117010. PubMed PMID: 36000730.
21. Kouka M, Buentzel J, Kaftan H, Boeger D, Mueller AH, Wittig A, et al. Early Mortality among Patients with Head and Neck Cancer Diagnosed in

Thuringia, Germany, between 1996 and 2016-A Population-Based Study. Cancers (Basel). 2022;14(13). Epub 20220624. doi: 10.3390/cancers14133099. PubMed PMID: 35804871; PubMed Central PMCID: PMCPMC9264998.
22. CP DEON, Brito HO, RMG DAC, Brito LMO. Is There a Role for Sex Hormone Receptors in Head-and-neck Cancer? Links with HPV Infection and Prognosis. Anticancer Res. 2021;41(8):3707-16. doi: 10.21873/anticanres.15162. PubMed PMID: 34281829.
23. Grunow J, Rong C, Hischmann J, Zaoui K, Flechtenmacher C, Weber KJ, et al. Regulation of submaxillary gland androgen-regulated protein 3A via estrogen receptor 2 in radioresistant head and neck squamous cell carcinoma cells. J Exp Clin Cancer Res. 2017;36(1):25. Epub 20170206. doi: 10.1186/s13046-017-0496-2. PubMed PMID: 28166815; PubMed Central PMCID: PMCPMC5294868.
24. Verma A, Schwartz N, Cohen DJ, Patel V, Nageris B, Bachar G, et al. Loss of Estrogen Receptors is Associated with Increased Tumor Aggression in Laryngeal Squamous Cell Carcinoma. Sci Rep. 2020;10(1):4227. Epub 20200306. doi: 10.1038/s41598-020-60675-2. PubMed PMID: 32144339; PubMed Central PMCID: PMCPMC7060328.
25. Verma A, Schwartz N, Cohen DJ, Boyan BD, Schwartz Z. Estrogen signaling and estrogen receptors as prognostic indicators in laryngeal cancer. Steroids. 2019;152:108498. doi: https://doi.org/10.1016/j.steroids.2019.108498.
26. Hamilton SN, Tran E, Berthelet E, Wu J, Olson R. Early (90-day) mortality after radical radiotherapy for head and neck squamous cell carcinoma: A population-based analysis. Head Neck. 2018;40(11):2432-40. Epub 20181008. doi: 10.1002/hed.25352. PubMed PMID: 30295975.
27. Datema FR, Ferrier MB, van der Schroeff MP, Baatenburg de Jong RJ. Impact of comorbidity on short-term mortality and overall survival of head and neck cancer patients. Head Neck. 2010;32(6):728-36. doi: 10.1002/hed.21245. PubMed PMID: 19827120.
28. Jakobsen KK, Gronhoj C, Jensen DH, Karnov KKS, Agander TK, Specht L, et al. Increasing incidence and survival of head and neck cancers in Denmark: a nation-wide study from 1980 to 2014. Acta Oncol.

2018;57(9):1143-51. Epub 20180215. doi: 10.1080/0284186X.2018.1438657. PubMed PMID: 29447088.
29. Massa ST, Osazuwa-Peters N, Christopher KM, Arnold LD, Schootman M, Walker RJ, et al. Competing causes of death in the head and neck cancer population. Oral Oncol. 2017;65:8-15. doi: 10.1016/j.oraloncology.2016.12.006. PubMed PMID: 28109473.
30. Cancercentrum. Nationellt vårdprogram huvud- och halscancer [Internet]: Regionala Cancercentrum i samverkan; 2015 [updated 15-08-25; cited 2017 december 4]. Available from: https://www.cancercentrum.se/samverkan/cancerdiagnoser/huvud-och-hals/vardprogram/gallande-vardprogram/.
31. Maxwell JH, Grandis JR, Ferris RL. HPV-Associated Head and Neck Cancer: Unique Features of Epidemiology and Clinical Management. Annu Rev Med. 2016;67:91-101. Epub 2015/09/04. doi: 10.1146/annurev-med-051914-021907. PubMed PMID: 26332002; PubMed Central PMCID: PMCPMC5242186.
32. Lechner M, Liu J, Masterson L, Fenton TR. HPV-associated oropharyngeal cancer: epidemiology, molecular biology and clinical management. Nat Rev Clin Oncol. 2022;19(5):306-27. Epub 20220201. doi: 10.1038/s41571-022-00603-7. PubMed PMID: 35105976; PubMed Central PMCID: PMCPMC8805140.
33. Lorenzoni V, Chaturvedi AK, Vignat J, Laversanne M, Bray F, Vaccarella S. The Current Burden of Oropharyngeal Cancer: A Global Assessment Based on GLOBOCAN 2020. Cancer Epidemiol Biomarkers Prev. 2022;31(11):2054-62. doi: 10.1158/1055-9965.EPI-22-0642. PubMed PMID: 36173880.
34. Mehanna H, Beech T, Nicholson T, El-Hariry I, McConkey C, Paleri V, et al. Prevalence of human papillomavirus in oropharyngeal and nonoropharyngeal head and neck cancer--systematic review and meta-analysis of trends by time and region. Head Neck. 2013;35(5):747-55. Epub 20120120. doi: 10.1002/hed.22015. PubMed PMID: 22267298.
35. Lydiatt WM, Patel SG, O'Sullivan B, Brandwein MS, Ridge JA, Migliacci JC, et al. Head and Neck cancers-major changes in the American Joint Committee on cancer eighth edition cancer staging manual. CA

Cancer J Clin. 2017;67(2):122-37. Epub 20170127. doi: 10.3322/caac.21389. PubMed PMID: 28128848.
36. Ramström L, Borland R, Wikmans T. Patterns of Smoking and Snus Use in Sweden: Implications for Public Health. Int J Environ Res Public Health. 2016;13(11). Epub 20161109. doi: 10.3390/ijerph13111110. PubMed PMID: 27834883; PubMed Central PMCID: PMCPMC5129320.
37. Folkhälsomyndigheten. Vuxnas bruk av tobaks- och nikotinprodukter www.folkhälsomyndigheten.se: Folkhälsomyndigheten; 2022 [cited 2023 230216]. Available from: https://www.folkhalsomyndigheten.se/livsvillkor-levnadsvanor/andts/utveckling-inom-andts-anvandning-och-ohalsa/anvandning/anvandning-av-tobaks-och-nikotinprodukter/vuxnas-bruk-av-tobaks--och-nikotinprodukter.
38. Folkhälsomyndigheten. Tobaksrökning, daglig [Internet] Stockholm: Folkhälsomyndigheten; 2022 [cited 2023 230305]. Available from: https://www.folkhalsomyndigheten.se/folkhalsorapportering-statistik/tolkad-rapportering/folkhalsans-utveckling/resultat/levnadsvanor/tobaksrokning-daglig/.
39. Gebre-Medhin M, Brun E, Engström P, Cange HH, Hammarstedt-Nordenvall L, Reizenstein J, et al. ARTSCAN III: A Randomized Phase III Study Comparing Chemoradiotherapy With Cisplatin Versus Cetuximab in Patients With Locoregionally Advanced Head and Neck Squamous Cell Cancer. Journal of Clinical Oncology. 2021;39(1):38-47. doi: 10.1200/jco.20.02072. PubMed PMID: 33052757.
40. Gaubatz ME, Bukatko AR, Simpson MC, Polednik KM, Adjei Boakye E, Varvares MA, et al. Racial and socioeconomic disparities associated with 90-day mortality among patients with head and neck cancer in the United States. Oral Oncol. 2019;89:95-101. Epub 20181229. doi: 10.1016/j.oraloncology.2018.12.023. PubMed PMID: 30732966.
41. Rieke K, Schmid KK, Lydiatt W, Houfek J, Boilesen E, Watanabe-Galloway S. Depression and survival in head and neck cancer patients. Oral Oncol. 2017;65:76-82. doi:

10.1016/j.oraloncology.2016.12.014. PubMed PMID: 28109472.

Figure 1

Click here to access/download;Figure;Figure1.pdf

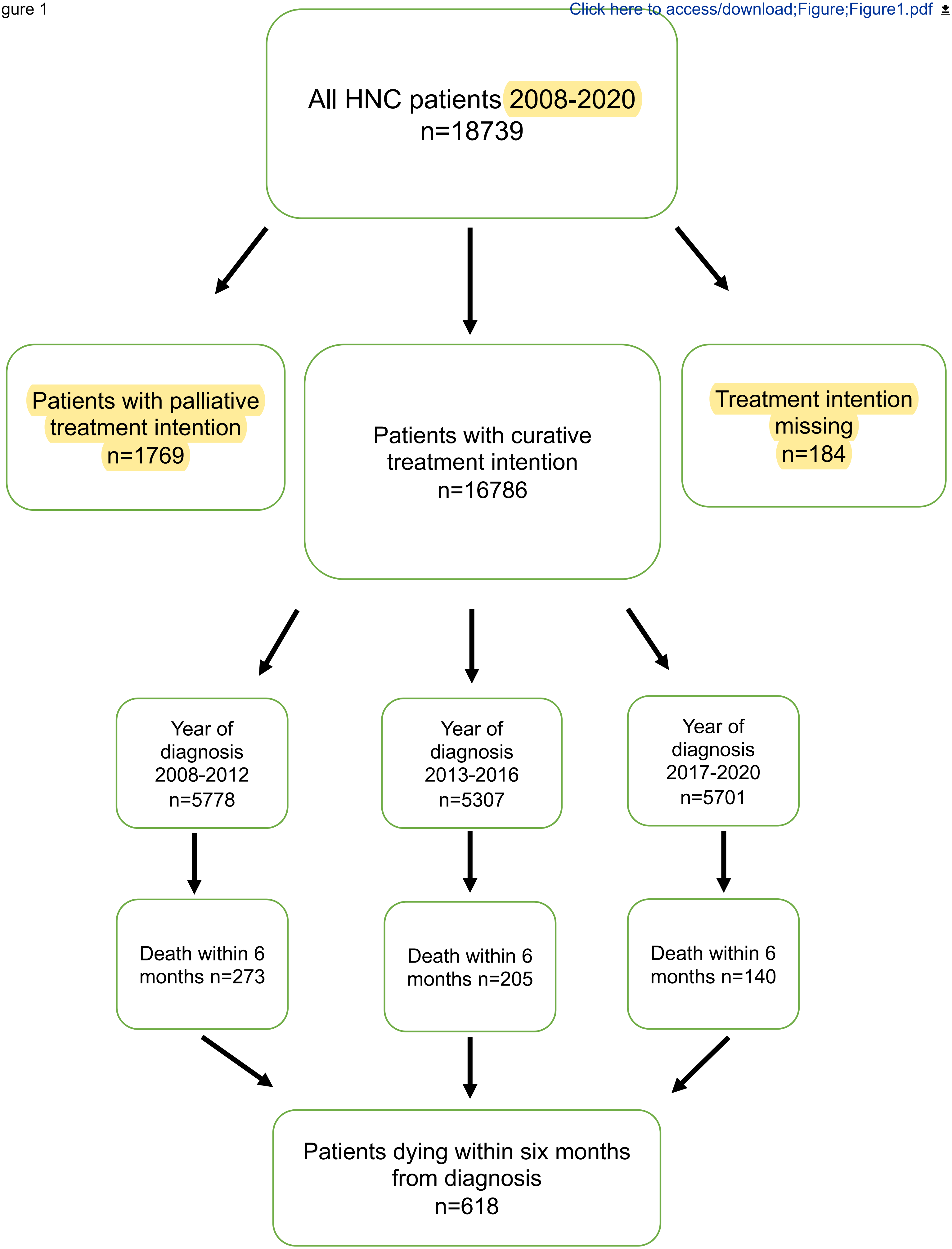

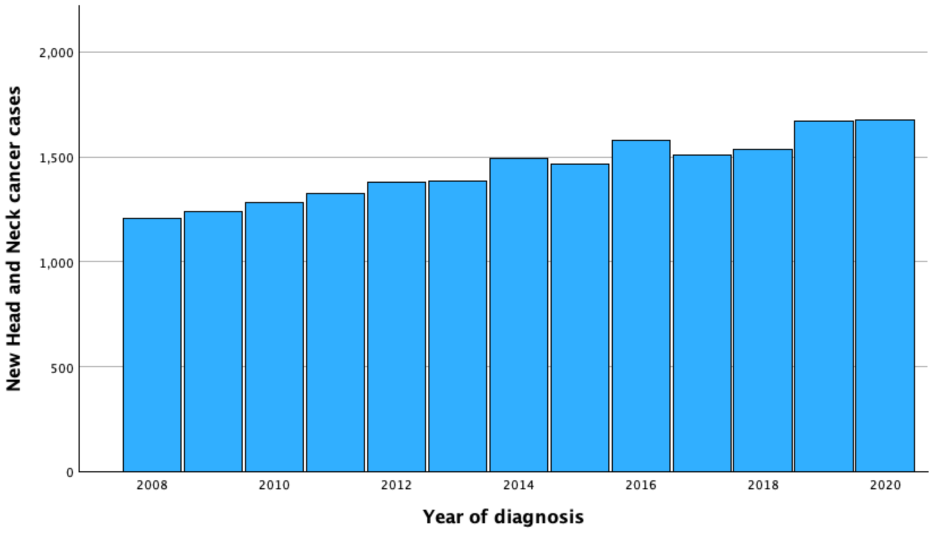

Figure 2a

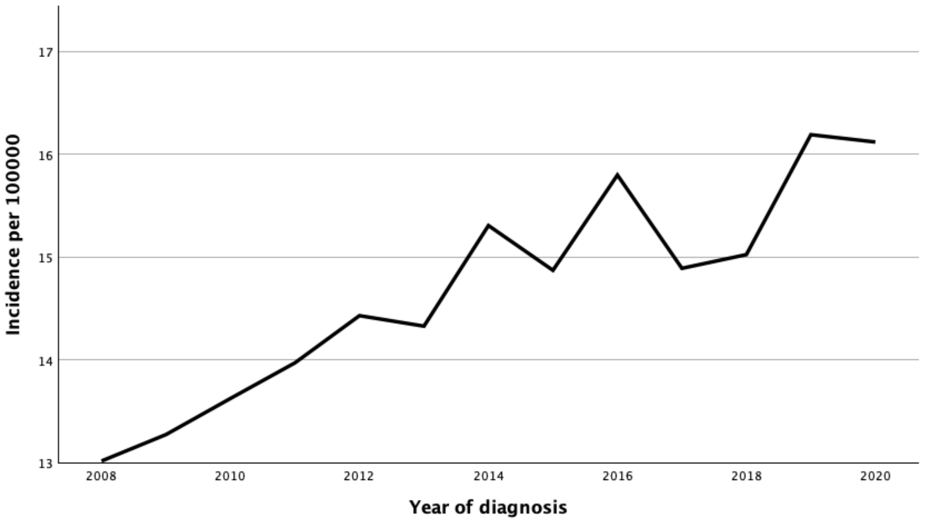

Figure 2b

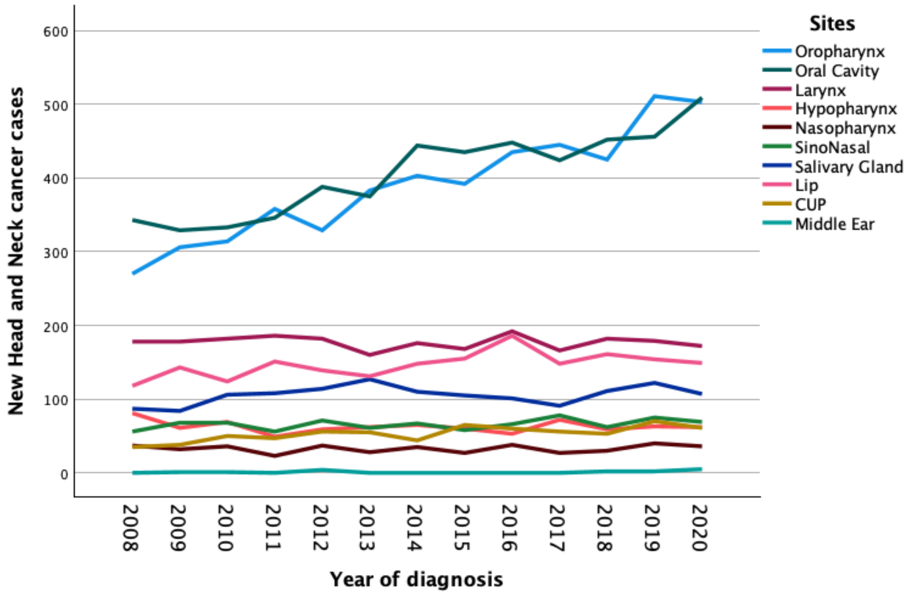

Figure 2c

Click here to access/download;Figure;Figure 3.pdf

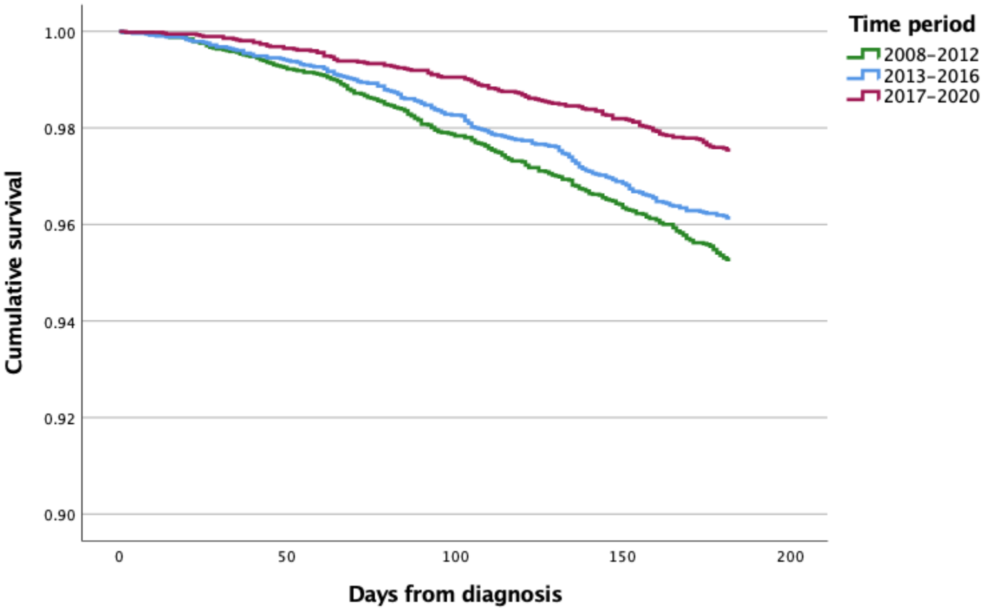

Figure 3

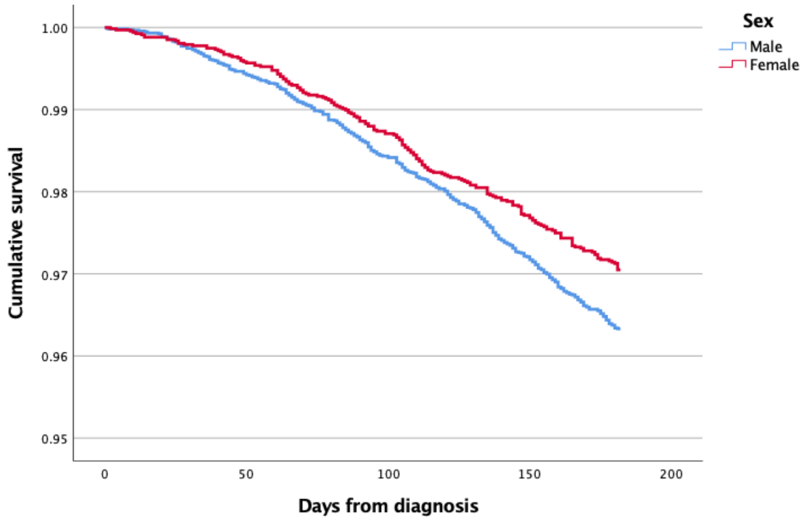

Figure 4a

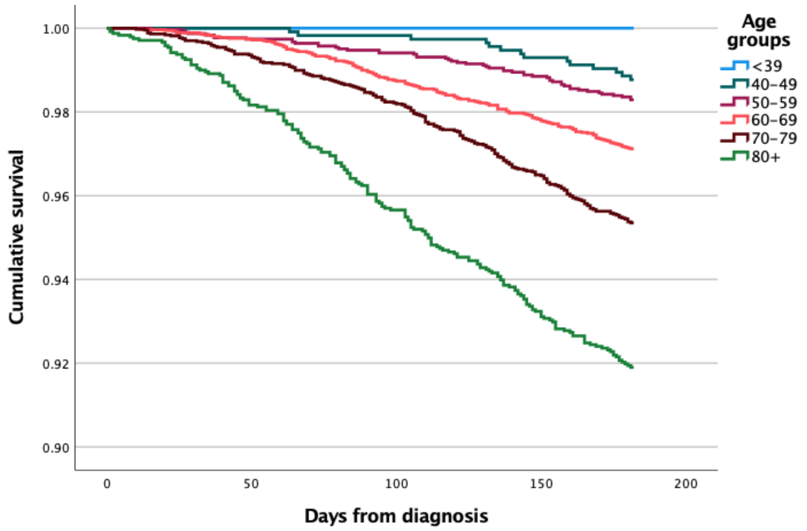

Figure 4b

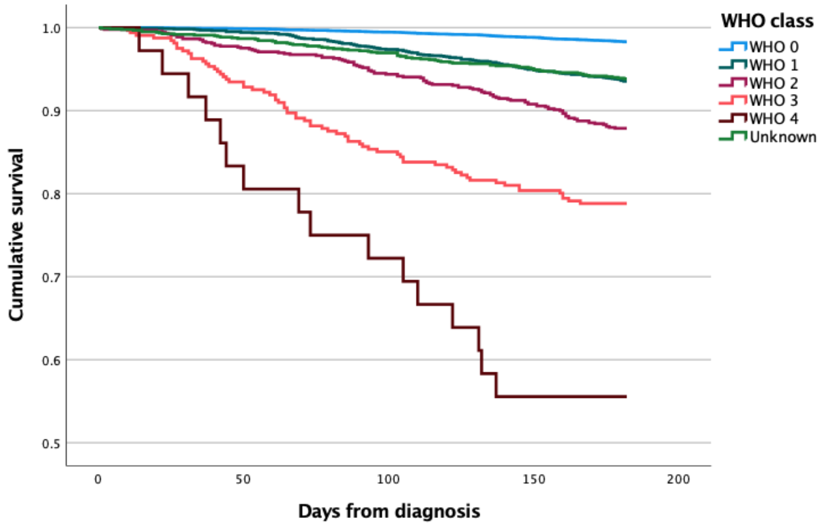

Figure 4c
